# A Comprehensive Experimental, Simulation, and Characterization Mechanical Analysis of Ecoflex and Its Formulation Under Uniaxial Testing

**DOI:** 10.3390/ma18133037

**Published:** 2025-06-26

**Authors:** Ranjith Janardhana, Fazli Akram, Zeynel Guler, Akanksha Adaval, Nathan Jackson

**Affiliations:** 1 Center for High Technology Materials, The University of New Mexico, Albuquerque, NM 87106, USAzeynelguler@unm.edu (Z.G.); aadaval06@unm.edu (A.A.); 2 The Department of Mechanical Engineering, The University of New Mexico, Albuquerque, NM 87131, USA; 3 The Nanoscience and Microsystems Engineering, The University of New Mexico, Albuquerque, NM 87131, USA

**Keywords:** Ecoflex, artificial skin, finite element modeling, wearable sensor, self-cleaning surface, silicone elastomer

## Abstract

The current study focuses on the manufacturing and characterization of various forms of Ecoflex and their composites to improve the mechanical properties and surface texture, specifically for use in wearable sensors and electronic skin applications. Various types of Ecoflex elastomers were mixed to form blended composite materials, which could be used to tune the mechanical properties. Experimental and simulation methods were conducted to understand the mechanical behavior and material properties of the manufactured samples under large deformation (1200% strain) by various dynamic loading conditions. Further, the surface conditions of specimens were analyzed and evaluated using scanning electron microscopy and contact angle goniometer. The Yeoh model reasonably predicts the viscoelastic and hysteresis behavior of Ecoflex and its composites in accordance with the experimental data for small and large strain. The surface smoothness and moisture-resistant properties of the material surface were enhanced up to a contact angle of 127° (maximum) by adding x = 15 wt% of surface tension diffusers, with a slight compromise in stretchability. This comprehensive investigation and database of Ecoflex–Ecoflex composite can guide and help researchers in selecting and applying the most appropriate Ecoflex/blended solutions for a specific application, while providing insight into the mechanics of materials of blended materials.

## 1. Introduction

Silicone elastomers have been extensively researched due to their unique physical properties, ease of manufacturing, and high reproducibility, which has led to a wide range of applications, including biomedical [1,2,3,4], aerospace [5,6], electronics [7,8,9], cosmetics [10,11,12], and textile [13,14]. These elastomers exhibit excellent characteristics such as biocompatibility, ease in manufacturing, relatively low cost, thermal stability, chemical resistance, tunability, and high flexibility [15,16,17,18,19,20]. Synthesis of nanoparticles into the elastomers can be used to enhance performance or add functionality to the material such as electrical conductivity, magnetic properties, and piezoelectric properties [21,22,23,24,25,26]. These multifunctional elastomer composites have resulted in the development of wearable sensors, flexible electronics [27], stretchable electronic skin (e-skin) [28], and mechanical sensors [29]. Silicone elastomers are utilized as a flexible/stretchable substrate (lower stiffness) to create actuators and sensors for soft robotic applications [30,31]. Additionally, biomedical devices such as dialysis membranes [32], artificial skin [33], prosthetics, and arterial phantoms [34] were developed by using elastomers due to their biocompatibility and chemical resistance. Ga-based liquid metal has been embedded in the elastomer to develop a stretchable monopole antenna and coaxial phase shifter [35,36,37] and various other devices [38]. Furthermore, Zhou et al. and Jeong et al. demonstrated the feasibility of creating a high power density stretchable energy harvester for smart material applications [39,40,41].

However, stretchable electromechanical sensors typically require the ability to withstand large strain (>800%) under repetitive loading cycles [42,43]. These sensors need to be soft and flexible with higher tear strength and lower stiffness materials. Similarly, infused polymer matrix wearable sensors and health monitoring sensors involve the utilization of ultra-soft stretchable and high-sensitivity substrates to detect human body motion [44]. Environmental stressors and cyclic loading conditions influence the effective functioning of the soft energy harvester [45]. In addition, the use of artificial e-skin on the human body is needed to cope with the complex human function with good adhesion, hydrophobicity, adaptability to rapid deformation, and durability. Young’s modulus of human skin varies with age, skin texture, gender, and location of the human body (5 kPa–140 MPa), which are significant factors for the design of electromechanical sensors or the development of artificial human skin [46,47], along with environmental conditions. The combination of the above characteristics such as flexibility, stretchability, durability, environmental stability, adaptability to dynamic loading conditions, and moisture resistance is necessary for the development of a wide range of flexible e-skin sensors or energy harvesters. Regardless, key challenges exist in the selection of elastomers based on their application to produce the desired performance.

Ecoflex is a silicone elastomer with relatively low stiffness (5 kPa–80 kPa), highly flexible and deformable, and biocompatibility, and it is adaptable to dynamic loading and environmental conditions. Ecoflex is prepared by crosslinking the platinum-based catalyst with the base component that is typically cured at room temperature, which forms a polymer chain structure. The softness of Ecoflex is specified by its shore hardness, which typically ranges from 000-34 (Gel2) to 00-50. Recent work highlighted that the mechanical behavior and stiffness of these elastomers vary greatly under large strain (>500%) irrespective of shore hardness [17]. Additionally, micro-scale Ecoflex samples become harder and stiffer under uniaxial testing, which affects the performance of microelectromechanical (MEMS) devices [48].The excellent tunability properties of Ecoflex have been demonstrated to improve the softness and stretchability when combined with other elastomers such as Sylgard 184, Sylgard 186, and Solaris [15,49,50]. Previous research conducted on the blending of Ecoflex–Ecoflex illustrated an enhancement in the ultimate strength and deformability of the material [48]. These improvements in the mechanical characteristics by blending the Ecoflex–Ecoflex mixture necessitate further investigations to explore the tunability of its properties for the desired applications such as developing artificial human skin or mountable flexible sensors on the skin. Moreover, human skin is a complex, highly resistive viscoelastic material that is significantly influenced by factors such as strain rate, cyclic loading, relaxation test, stretching, and hydration [51].

In this paper, we investigate the mechanical properties of Ecoflex elastomers with varying shore hardness values along with creating blended composites of Ecoflex to determine the impacts it has on the mechanical properties. Ecoflex with shore hardness 00-10, 00-20, 00-30, Gel2 (000-34) and its combination mixtures with respect to different weight ratios were investigated. Despite the selected Ecoflex samples being prepared in a similar fashion, the mechanical response/failure of these elastomers is considerably affected due to the variation in the polymer structure induced by the reaction of prepolymer and crosslinker [17]. For example, the result shows that widely used Ecoflex 00-30 fails under continuous five loading–unloading cycles and stress relaxation tests compared to the other Ecoflex formulation. Due to this variability, the current study comprehensively analyzes the material behavior of Ecoflex by subjecting the prepared samples to various real-world conditions. Further, the relationship between shore hardness, peak strength, and Young’s modulus was interpolated based on the experimental data set. Additionally, the finite element model (FEM) was developed to predict the material properties that can be infused in the development of a particular application. The scanning electron microscopy (SEM) method is used to examine the morphological structure of pristine and fractured samples. This work establishes the complete database for the researchers in the selection of different formulations of the Ecoflex–Ecoflex mixture for the advancement of smart material devices.

## 2. Materials and Methods

### 2.1. Materials

Four distinct shore hardnesses of Ecoflex 00-10, 00-20, 00-30, and Gel2 (000-34) from Smooth-On Inc., Macungie, PA, USA, were utilized for this study. Ease Release 200 aerosol spray (Mann Release Technology, Macungie, PA, USA) was applied to the mold as a release agent. Customized molds were created using a 3D printer (Ultimaker S5, Zaltbommel, The Netherlands), 3M 71601 blue squeegee, and a mixing container of diameter 35 mm for leveling and mixing samples, respectively. SLIDE^TM^ Liquid surface tension diffuser (ST-Diffuser) from Smooth-On Inc., USA, was added when specifically mentioned to alter the surface properties and the hydrophobicity of the elastomer.

### 2.2. Sample Preparation

**Figure 1:**
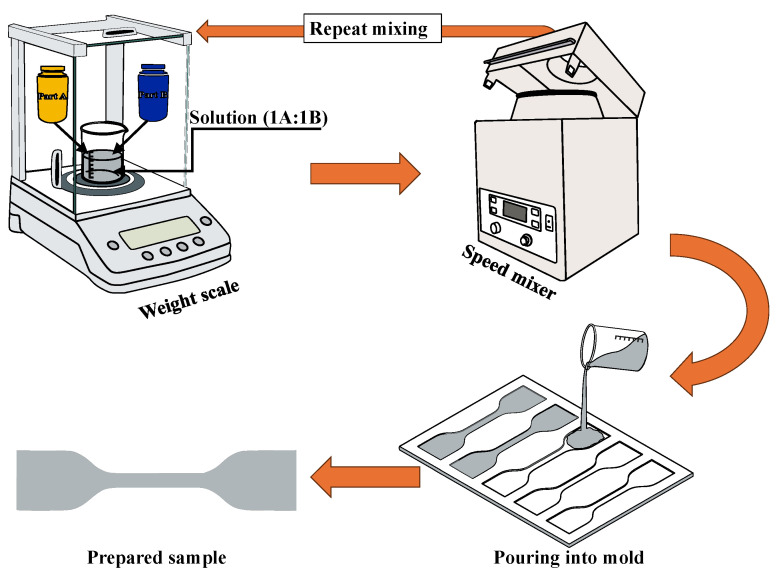
Schematic representation of sample preparation cycle.

**Figure 2:**
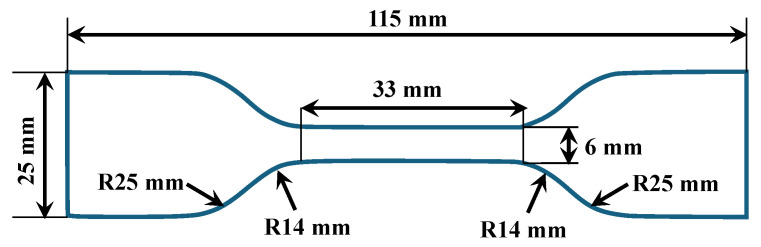
Dimensions of mold prepared as per ASTM-D412-C.

The sample preparation steps are illustrated in schematic Figure 1 [52]. First, two parts of liquid components, the base (Part A) and crosslinker (Part B) of Ecoflex, were weighed in a container with a precision balance scale (MTI Corporation, Richmond, CA, USA) in a weight ratio 1 (Part A):1 (Part B). Then the sample was mixed thoroughly in a speed mixer (FlackTek, Inc., Landrum, SC, USA) at a speed of 3000 rpm for 1 min. Based on different speed rates, it was observed that mixing the sample at 3000 rpm resulted in minimizing the microbubble formation. The combined solution sample was immediately poured into a customized mold that had been coated with a release agent approximately 30 min before. A doctor’s blade method technique was followed to remove excess liquid from the mold and establish uniform thickness of the specimen. All samples were allowed to cure for 24 h at room temperature before demolding. Since the mixtures had different curing durations, a 24 h curing time was chosen to ensure that all samples were fully cured before removal from the mold. The dimensions of the manufactured dogbone-shaped mold were in accordance with the American Society for Testing of Materials (ASTM-D412-C) standards [53], as shown in Figure 2.

The same steps mentioned above were used to produce samples from Ecoflex 00-10, 00-20, 00-30, and Gel2, which were considered as a “base liquid”. Similar procedures were followed to create Ecoflex–Ecoflex blended samples by mixing pairs, triplets, and quadruples of base liquids, and for convenience, they were referred to as “two type mixing”, “three type mixing”, and “four type mixing”, respectively. After each stage of combining different Ecoflex liquids, the resulting solutions were mixed in the speed mixer. The total weight of the final solution considered in a mixing container was 22 g. Weight ratios for mixing different Ecoflex shore hardness liquids were calculated based on the concentration, 25%, 50%, 75% and 100%, as tabulated in Supplementary Material Table S2. However, Part A and Part B were maintained in a weight ratio of 1:1 for each Ecoflex liquid mixture. All the samples were identically prepared, blended, molded, cured, and demolded. Hereafter, Ecoflex composition solutions of 00-10, 00-20, 00-30, and Gel2 are mentioned as 0010:0020:0030:Gel2 for simplicity.

Although the combining process lasted 20 min for four different Ecoflex mixing ratios (0010:0020:0030:Gel2), it was observed that the lower viscosity flow of the solutions with ratios 1:4:1:1, 1:1:4:1, 1:1:1:3, and 3:3:3:2 was insufficient. The pot life of Gel2 and 0010 were 20 and 30 min, respectively, so high concentrations of these two blended materials had issues with molding. The pot life of the final solution decreased especially for 3:3:4:3, 4:1:1:1, 2:2:2:3, and 2:3:2:2, which resulted in difficultly in pouring the sample into the mold. Potential causes of the reduced pot life of the final solution include the concentration of the various Ecoflex liquids, prolonged combining durations, or temperature rise in the solution in a container during the mixing cycles in a speed mixer.

### 2.3. Experimental Setup and Characterization

**Figure 3:**
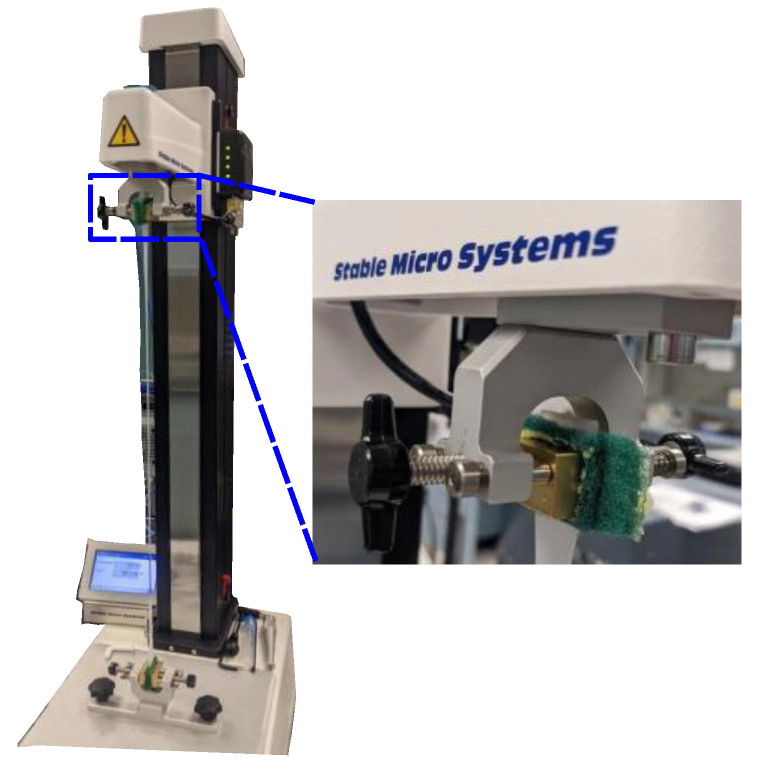
Universal texture analysis testing system with scour pads for metallic grip.

All the fabricated samples were subjected to uniaxial tensile test using universal texture analysis equipment (Stable Mirco Systems, Godalming, UK) with a 5 kg load cell at room temperature. Various types of end strips such as different grits of sandpaper, thick paper, and scour pads (Scotch-Brite, 3M, St. Paul, MN, USA) were investigated to prevent slippage of samples from metallic grip during stretching. However, scour pads were effectively bonded between samples and metallic grip, which avoided the slippage of samples, as shown in Figure 3.

Ecoflex 0010, 0020, 0030, and Gel2 (base liquids) and its mixture formulation 2:1 (two mixing ratio), 2:1:2 (three mixing ratio), and 2:1:2:2 (four mixing ratio) were considered as “base samples”. The mechanical behavior of the base samples was characterized by strain rate, cyclic test, stress relaxation, Mullins effect, specimen age condition, and SEM. Stickiness of the Ecoflex surface was measured using a TA-3 probe (Stable Mirco Systems, Godalming, UK) (diameter of 25.4 mm) with universal texture analysis equipment. Contact angle goniometer (Ossila, Sheffield, UK) and digital shore hardness durometer (Model HT-6510, Grainger, NY, USA) were utilized for assessing the hydrophobicity and shore hardness of the material surface. Shore hardness was measured by manually pressing the durometer onto the surface of each sample. A total of 10 readings were taken at different locations and the average was reported.

## 3. Results and Discussion

### 3.1. Reproducibility

**Figure 4:**
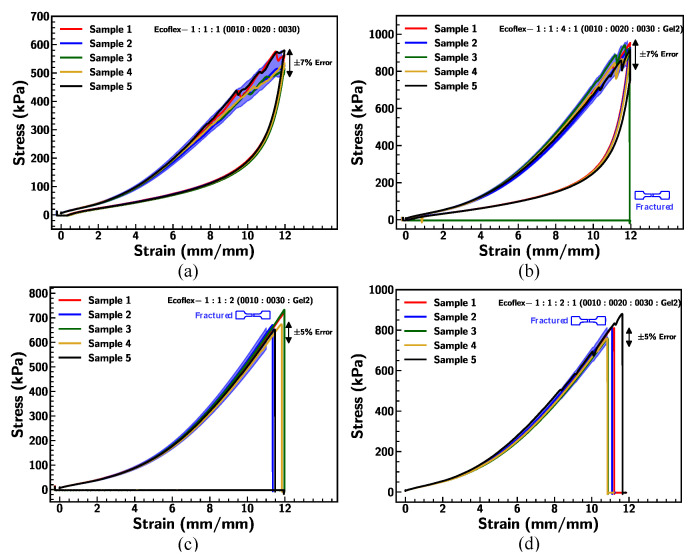
Stress–strain curve of Ecoflex: (**a**) 1:1:1 (0010:0020:0030), (**b**) 1:1:4:1 (0010:0020:0030:Gel2), (**c**) 1:1:2 (0010:0030:Gel2), and (**d**) 1:1:2:1 (0010:0020:0030:Gel2) at 0.1/s strain rate with dogbone illustrating breaking point.

**Figure 5:**
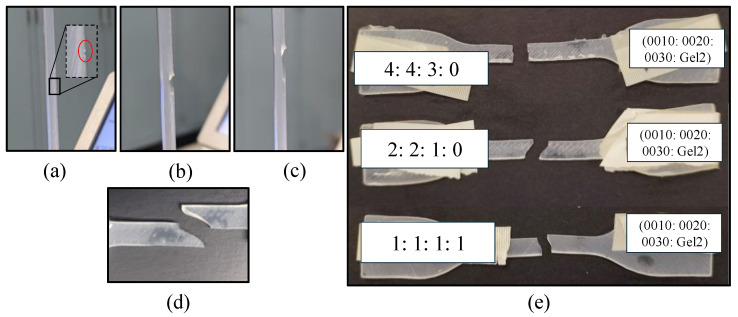
(**a**–**d**) Crack propagation during deformation under stretch; (**e**) fractured samples.

In detail, the consistency of the result produced by the prepared specimen under uniaxial tensile test conditions was demonstrated in Figure 4. Each fabricated Ecoflex mixture ratio consists of five samples and the results were evaluated by stretching up to 1200% strain. In Figure 4a, five samples of three type mixing ratio 1:1:1 (0010:0020:0030) were subjected to cyclic loading–unloading at 0.1/s strain rate, producing the hysteresis loop with a ±7% margin of error. The loading and unloading path of four type mixing ratio 1:1:4:1 (0010:0020:003:Gel2) was found to be within 7% error where sample 3 was fractured at 1200% strain. The results of three type mixing ratio 1:1:2 (0010:0030:Gel2) and four type mixing ratio 1:1:2:1 (0010:0020:0030:Gel2) (Figure 4c and Figure 5d) exhibit good repeatability of the stress–strain curve with a 5% error. These outcomes display the consistency in the mechanical behavior of the batch samples that were generated from uniaxial tensile testing equipment.

Additionally, it was observed that the failure of the various samples was occurring near the middle of the gauge length (Figure 5e), indicating that the samples were not slipping under a large deformation. Figure 5a–d demonstrates that the presence of a notched crack leads to an oblique fracture of the material. However, failure of the samples may occur due to the variation in the formation of the polymer chain, presence of gas entrapment (air bubbles), contamination, micro-cracks, and position of the samples [34], which resulted in different modes of fracture, as represented in Figure 5e.

**Figure 6:**
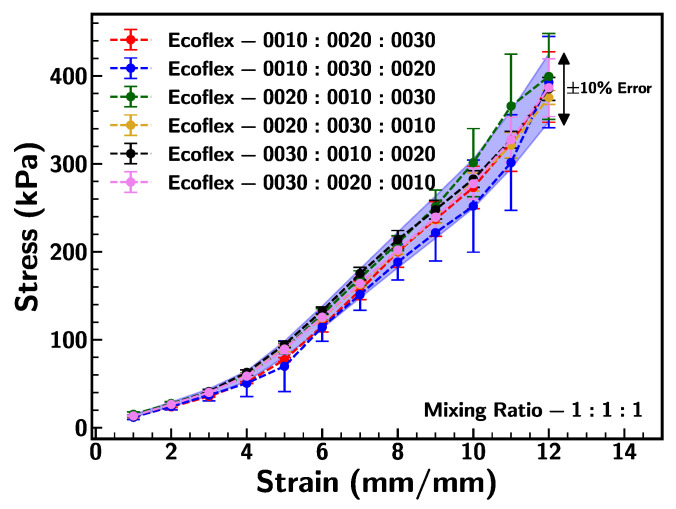
Comparison of stress–strain curve for mixing ratio 1:1:1 (0010:0020:0030) with varying mixing sequence at 0.1/s strain rate.

Furthermore, the effect of the mixing sequence on the mechanical behavior of the sample was analyzed by considering three type mixing ratio 1:1:1 (0010:0020:0030). All six possible mixing combinations of different samples were tested under uniaxial testing. Figure 6 represents the comparison of stress–strain behavior and the mean error in the results was within a 10% range. This suggests that the sequence of mixing the Ecoflex of different shore hardnesses has less effect on the mechanical behavior. Therefore, the effect of mixing sequences for other blended ratios was not considered within the scope of this study.

### 3.2. Young’s Modulus and Peak Stress

Young’s modulus of the prepared samples was evaluated at 100% strain within the linear region of the stress–strain curve, as all samples retained their original shape for more than five cycles of loading–unloading at this strain [48]. Further, stretching of the Ecoflex (>200%) undergoes continuous deformation with steep increments in the resistance from the material. Hence, tensile stress was evaluated at a specific location of strain from 100 to 500% due to the nonlinear behavior of the samples [34,54]. Peak stress refers to the maximum amount of stress experienced by the Ecoflex under strain at 1200%. Supplementary Figure S1 shows the variation in peak stress and Young’s modulus under strain 1200% for all the formulations of the Ecoflex ratio. Similarly, the boxplot (Supplementary Figure S2) represents the extension of the blend solution samples tested under strain 1200%, with most specimens having stretchability greater than 1200% strain. Ecoflex formulations 3:2:2:0, 2:3:3:3, and 0:0:2:3 (0010:0020:0030:Gel2) have the least stretchability of 950% strain. These results signify that Ecoflex can be the ideal material for the stretchable sensor and e-skin applications.

### 3.3. Strain Rate

**Figure 7:**
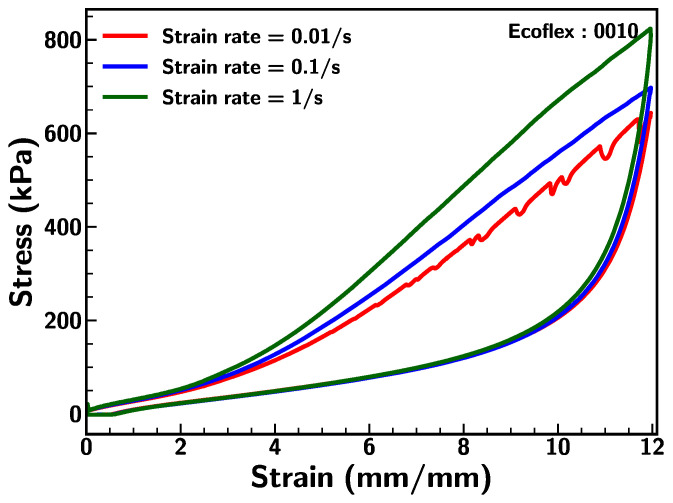
Stress–strain curve under varying strain rates of 0.01/s, 0.1/s and 1/s for Ecoflex 0010.

Base Ecoflex samples were subjected to different stretching rates to examine the strain rate dependency. Figure 7 depicts the mechanical behavior of Ecoflex 0010 under strain rates of 0.01/s, 0.1/s, and 1/s. The stress–strain relationship indicates that the strain rate effect was insignificant under initial strain (<200%); however, specimen 0010 exhibits significant resistance for a higher strain rate under larger elongation (>200%). This suggests that the stretching rate influences the mechanical behavior of the specimen. However, the tested sample exhibited an uneven material response, which can be attributed to the propagation of micro-cracks or the expansion of micro-voids at higher deformation levels (>700% strain), due to increased data acquisition resolution from the tensile machine at a slower strain rate of 0.01/s. This can also be caused by the fast recovery of the material, which is more prominent at low strain rates.

**Figure 8:**
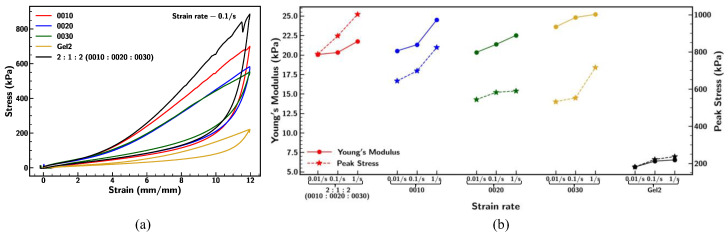
Comparison of (**a**) stress–strain curve under strain rate of 0.1/s, (**b**) Young’s modulus and peak stress under different strain rates of 0.01/s, 0.1/s, and 1/s for Ecoflex 0010, 0020, 0030, Gel2, and mixing ratio 2:1:2 (0010:0020:0030).

Figure 8 displays the comparison of stress–strain relationships, measured values of Young’s modulus, and peak stress of different base samples 0010, 0020, 0030, Gel2, and 2:1:2 (0010:0020:0030) at a strain rate of 0.1/s. The initial Young’s modulus was found to be minimum for Gel2 (~6kPa) and highest for Ecoflex 0030 (~25 kPa). These results show that the elastomers with a higher shore hardness were stiffer [34] under small strain. The methods used to manufacture Ecoflex can significantly affect Young’s modulus [52].

However, it was evident from Figure 8a that lower shore hardness Ecoflex such as 0010, 0020, and mixture 2:1:2 (measured shore hardness = 16) becomes stiffer under large strain. Similar behavior was observed for vulcanized rubber of higher stiffness subjected to large strain [55]. This indicates that after a certain threshold (strain > 200%), the mechanical behavior of the flexible samples significantly differs by exhibiting higher resistance to the larger deformation due to strain hardening. These changes in stress–strain behavior correspond to the polymer chain alignment and crosslinking process in the molecular structure, which was considerably affected under large strain [34], despite mixing formulation 2:1:2 having similar initial stiffness with respect to 0010 and 0020. A comparison of strain rate dependency for different Ecoflex samples is demonstrated in Figure 8b. The result indicates that the samples behave more rigidly under the influence of a faster stretching rate.

**Figure 9:**
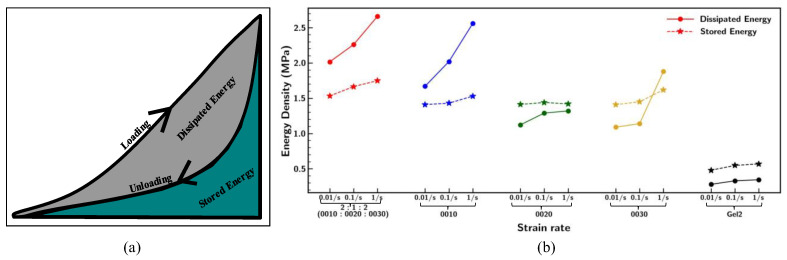
(**a**) Representation of energy density; (**b**) comparison of energy density under different strain rates of 0.01/s, 0.1/s, and 1/s for Ecoflex 0010, 0020, 0030, and Gel2 and mixing ratio 2:1:2 (0010:0020:0030).

Although Figure 8a shows different loading paths for base samples 0010, 0020, 0030, and 2:1:2, all the samples follow a similar unloading cycle path. This suggests that, despite variations in the loading behavior, the materials demonstrate comparable recovery characteristics upon unloading. This was further investigated by expressing the stress–strain curve in terms of energy density by bifurcating the loading–unloading cycle into dissipated and stored energy, as represented in Figure 9a. Total energy was computed by integrating the area inside the hysteresis loop and energy recovered under the unloading cycle [18], given by

(1) $$W_{t}=W_{d}+W_{s},$$

where $W_{t}$, $W_{d}$, and $W_{s}\;$are total energy density, dissipated energy density, and stored energy density, respectively.

The findings in Figure 9b display the increment in the hysteresis loop area by dissipating large amounts of energy with a faster stretching rate, which aligns with the pattern observed in Figure 8b. However, Gel2 samples showed the least changes to the deformation rate due to the high flexibility of the material. Although Ecoflex 0010, 0020, and 0030 and mixing ratio 2:1:2 had a comparable elastic stored energy density, the mixing ratio of 2:1:2 (0010:0020:0030) absorbs more energy by resisting larger deformation, which signifies the effect of complex polymer structure with respect to large strain.

### 3.4. Cyclic Loading

**Figure 10:**
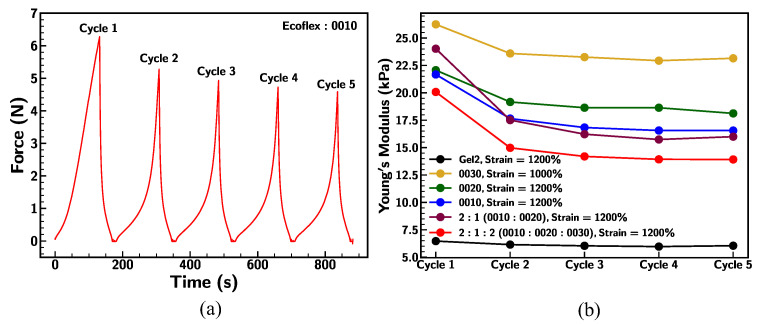
(**a**) Ecoflex 0010 under five cyclic loading–unloading; (**b**) comparison of Young’s modulus of base Ecoflex samples under five cyclic loading–unloading conditions at strain rate of 0.1/s.

Figure 10a demonstrates the reduction in the force under five loading–unloading stretching cycles. The peak force observed during each cycle decreases gradually, indicating a slight material softening. This softening may stem from internal molecular structural rearrangements, such as polymer chain reorientation or stress relaxation [56]. In detail, a comparative analysis of Young’s modulus for various Ecoflex samples over five cycles is presented in Figure 10b. A significant reduction in modulus was observed from Cycle 1 to Cycle 2, followed by a stabilization in subsequent cycles. Among the tested samples, Ecoflex 0030 shows the highest modulus, maintaining values above 23 kPa throughout the cycles, reflecting its superior stiffness and structural integrity. Ecoflex Gel2 exhibits the lowest modulus (~5 kPa), indicative of its softer and more flexible nature. The 2:1:2 mixture (0010:0020:0030) displays intermediate mechanical behavior, balancing stiffness and compliance. These findings highlight the ability to tune mechanical properties through specific formulations, enabling targeted applications based on stiffness and loading requirements.

**Figure 11:**
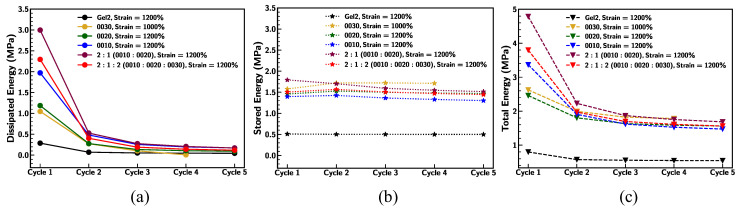
Comparison of (**a**) dissipated energy density, (**b**) stored energy density, and (**c**) total energy density of base Ecoflex samples under five cyclic loading–unloading conditions at strain rate of 0.1/s.

Further, the energy density of the base samples was evaluated under cyclic loading conditions and is outlined in Figure 11. The dissipated energy density sharply decreases from Cycle 1 to Cycle 2 and stabilizes thereafter for all samples. Gel2 has the lowest dissipated energy density across all cycles. The 2:1:2 blend and pristine 0010 sample exhibit the highest dissipated energy density in the first cycle, followed by stabilization at similar levels across subsequent cycles. Dissipated energy represents energy loss due to internal friction or material hysteresis during loading and unloading. The sharp decrease indicates a reduction in energy loss, likely due to polymer structural adjustments or material fatigue [57].

On the other hand, the stored energy density remains nearly constant across cycles for all samples. Ecoflex 0030 demonstrates the highest stored energy density, indicating higher stiffness. Gel2 has the lowest stored energy density, showing greater compliance. Stored energy density reflects the material’s ability to elastically store mechanical energy. The constant values across cycles imply minimal degradation in the elastic properties under cyclic loading. The higher stored energy for stiffer samples (e.g., 0030) suggests a correlation with their higher Young’s modulus, as seen in Figure 11b [58].

The total energy density decreases gradually from Cycle 1 to Cycle 5 for all samples, following a similar trend to the dissipated energy density in Panel a. The 2:1:2 blend and 0010 initially have the highest total energy density. Gel2 consistently exhibits the lowest total energy density across cycles. Total energy density includes both dissipated and stored energy. The decrease suggests that the material undergoes changes (e.g., polymer chain reorganization or microstructural fatigue), reducing its energy-handling capacity.

### 3.5. Stress Relaxation

**Figure 12:**
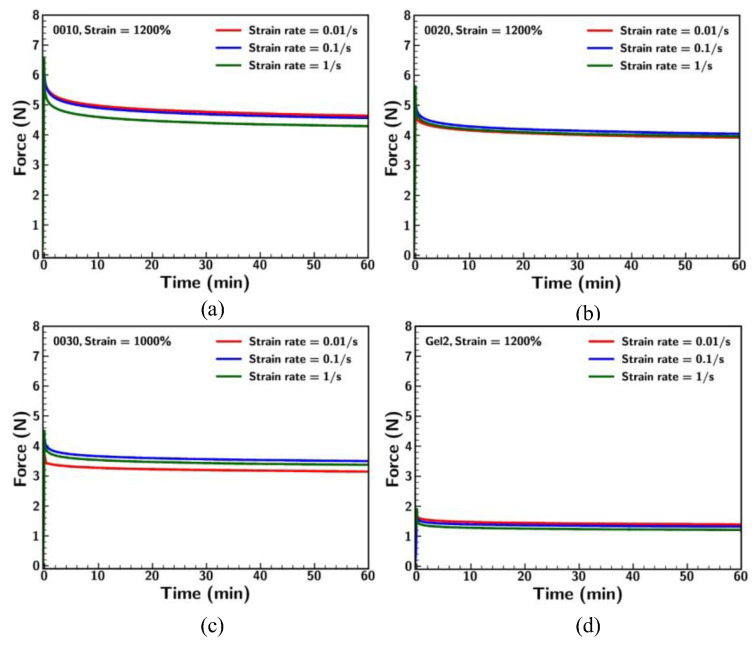
Comparison of stress relaxation for Ecoflex (**a**) 0010, (**b**) 0020, (**c**) 0030, and (**d**) Gel2 under different strain rates.

Stress relaxation experiments were conducted to investigate the viscoelastic properties of Ecoflex samples under different strain rates and conditions. These experiments provide an understanding of the durability and mechanical behavior of the material that is crucial for applications requiring sustained deformation. Figure 12 illustrates the stress relaxation behavior of the Ecoflex samples (0010, 0020, 0030, and Gel2) under different strain rates (0.01/s, 0.1/s, and 1/s) at a fixed strain of 1200%, except for Ecoflex 0030 (1000%). Ecoflex 0010 showed the highest initial stress across all strain rates, with a significant relaxation phase. The differences in strain rate were most evident in the first relaxation period, where higher strain rates (1/s) resulted in slightly higher initial forces. Nevertheless, long-term stress levels converged across all strain rates. Ecoflex 0020 demonstrated slightly lower initial forces than 0010 but displayed a similar relaxation trend. The influence of strain rate on long-term force was minimal. Ecoflex 0030 showed significantly lower initial forces compared to 0010 and 0020, with rapid relaxation and convergence across strain rates. Although 0030 showed higher stiffness (strain < 200%), the material exhibits softness under large stretching. It was observed that a few samples of 0030 failed under 1200% strain, highlighting the potential failure of Ecoflex 0030 over a long stretch condition duration. Gel2, which was the softest among all the materials, exhibited the lowest initial forces and the fastest relaxation, along with minimal dependence on strain rate. These results underline the role of large deformation and strain rate influencing the relaxation behavior of Ecoflex. Samples like Ecoflex 0010 retained higher stress over time, while softer materials like Gel2 exhibit rapid relaxation and demonstrate the risk of breakage of 0030 during long-term stretching [34].

**Figure 13:**
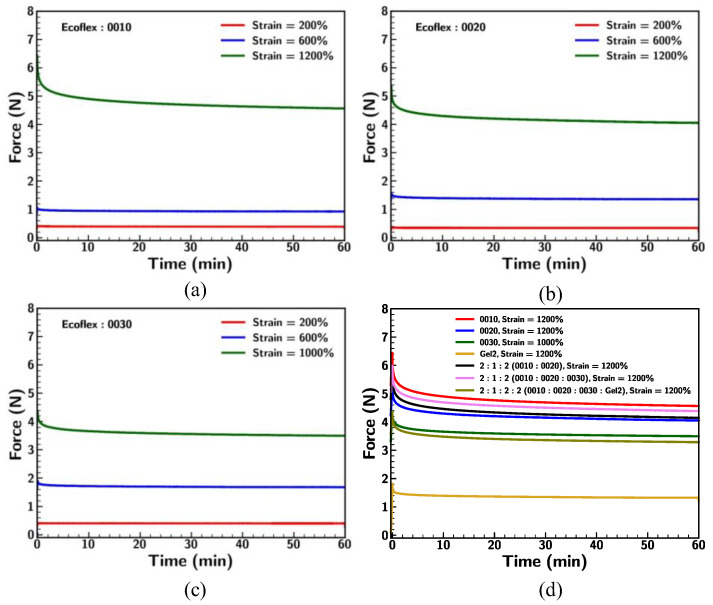
Comparison of stress relaxation for Ecoflex (**a**) 0010, (**b**) 0020, and (**c**) 0030 under different strain conditions at strain rate of 0.1/s; (**d**) comparison of base Ecoflex samples under stress relaxation condition at strain rate of 0.1/s.

Figure 13 and Table 1 compare the stress relaxation behavior of the Ecoflex samples under varying strain levels including 200%, 600%, and 1000% or 1200% at a constant strain rate of 0.1/s. The relaxation ratio was evaluated by comparing the initial force to the final force at the end of the 1 h period. For Ecoflex 0010, higher strains (1200%) resulted in higher initial stress and a slower rate of stabilization with a relaxation ratio of 29.1%. Lower strains (200% and 600%) showed lower stress responses and achieved immediate stress equilibrium with relaxation ratios of 3.8% and 12.7%, respectively. This indicates that stress relaxation increases with stretching. Ecoflex 0020 followed similar trends, with slightly lower initial stresses and a faster steady state than 0010. The result demonstrates that Ecoflex 0030 relaxed more rapidly, which signifies its softer mechanical properties under large strain in accordance with the earlier result. In Figure 13d, a comparison was conducted for base Ecoflex samples and their mixtures under stress relaxation at 1200% strain and a strain rate of 0.1/s. Ecoflex 0010 exhibited the highest initial stress and a high degree of relaxation, which is consistent with its stiffer nature for larger deformation. Gel2, the softest material, demonstrated the lowest stress response and the minimal relaxation rate (24.7%). The mixtures of Ecoflex samples (e.g., 2:1:2 (0010:0020:0030) exhibited intermediate relaxation profiles with lower stress relaxation. This suggests that combining materials allows for tuning stress relaxation properties, providing versatility in adapting materials for specific applications.

### 3.6. Mullin Effect

**Figure 14:**
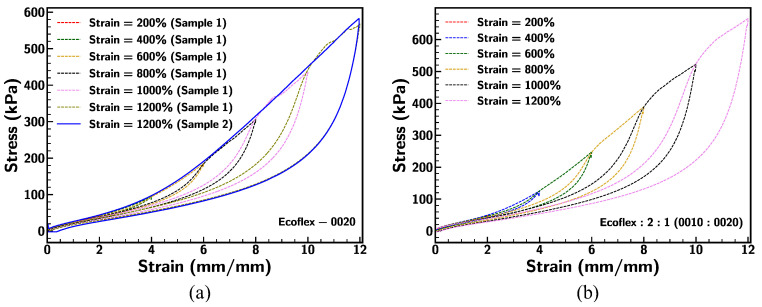
(**a**) Comparison of stress–strain behavior for Ecoflex 0020 with sample 1 under consecutive 200% to 1200% strain and sample 2 under 1200% strain only at strain rate of 0.1/s; (**b**) stress–strain curve under different strain for mixing ratio of Ecoflex 2:1 (0010:0020) at strain rate of 0.1/s.

Mullin’s effect, which is a stress-softening behavior observed in silicone elastomers after cyclic loading, was recorded for the base samples in this study. A comparison of the stress–strain relationship between sample 1 (cyclic loading–unloading condition at different strains) and sample 2 (cyclic loading–unloading condition at 1200% strain) is portrayed in Figure 14a. Ecoflex 0020 progressively loses its stiffness as the strain increases. Sample 1 shows the reduction in the stress up to the previous stretch level and regains its stress hardening in consecutive cycles following the same path as sample 2, demonstrating the viscoelastic behavior of the material [59]. Ecoflex 2:1 (0010:0020) shows similar nonlinear stress–strain behavior, with strain stiffening at higher strains. Stress levels were higher across all strains compared to pristine Ecoflex 0020, reflecting the influence of the stiffer Ecoflex 0010 component under larger deformation. At a strain of 1200%, the stress response was significantly greater than at lower strains, indicating enhanced stiffness due to the silicone composite.

**Figure 15:**
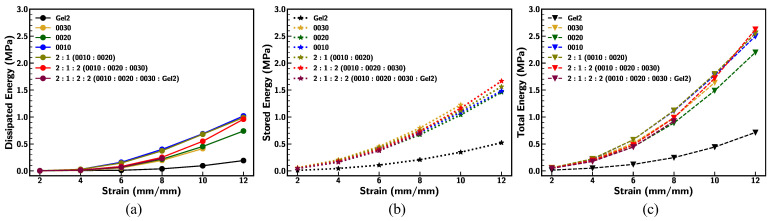
Comparison of (**a**) dissipated energy density, (**b**) stored energy density, and (**c**) total energy density of base Ecoflex samples under different stretching conditions at strain rate of 0.1/s.

Figure 15 illustrates the comparison of dissipated energy density (Figure 15a), stored energy density (Figure 15b), and total energy density (Figure 15c) for base Ecoflex samples under various stretching conditions at a strain rate of 0.1/s. The dissipated energy density, representing energy loss during deformation, shows a consistent increase with strain across all samples, with variations influenced by material composition. Similarly, the stored energy density, indicative of elastic energy retention, rises higher than dissipated energy at larger deformation (>500%). Differences between sample formulations reveal the impact of material modifications on energy dissipation and storage efficiency under dynamic loading conditions [60]. Among all, Ecoflex 0030 fails due to subsequent dynamic loading cycles (at strain 1200%), indicating that softening of the material led to failure under large strain.

### 3.7. Shore Hardness

The shore hardness (SH) of each prepared sample was evaluated using the shore hardness tester. Table 2 shows the average measured values of shore hardness for base Ecoflex samples. The measured shore hardness of Ecoflex 0010, 0020, and 0030 were in good agreement with the manufacture’s specifications. Further, the shore hardness of fabricated samples was evaluated and specifically explored the relationship between stiffness and peak stress using Pearson’s correlation (r) to fit the set of data.

(2) $$r_{xy}=\frac{\sum_{i=1}^{n}\left(x_{i}-\bar{x})(y-\bar{y}\right)}{\sqrt{\sum_{i=1}^{n}{\left(x_{i}-\bar{x}\right)}^{2}}\sqrt{\sum_{i=1}^{n}{\left(y_{i}-y\right)}^{2}}}$$

where *n* is the sample size, $x_{i}$ and $y_{i}$ represent the mean of each Ecoflex formulation, and $\bar{x}$ and $\bar{y}$ are the sample mean.

**Figure 16:**
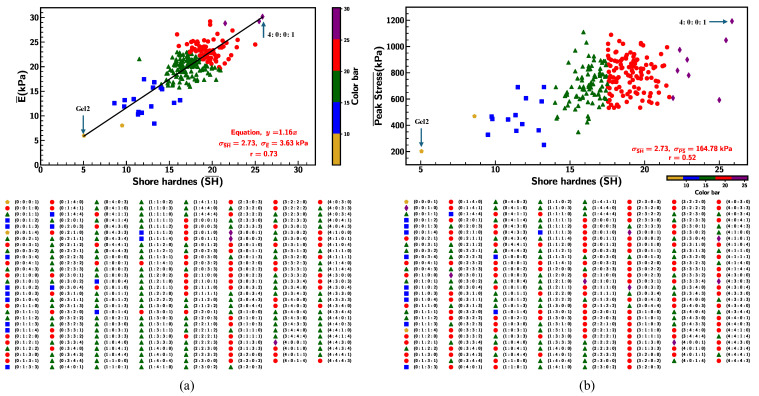
Correlation between (**a**) Young’s modulus and shore hardness, and (**b**) peak stress and shore hardness at strain rate of 0.1/s.

Figure 16 shows the correlation between shore hardness, Young’s modulus, and peak stress for different formulations of Ecoflex. A strong positive linear correlation (r = 0.73) exists between Young’s modulus and shore hardness, as represented in Figure 16a. Linear equation y=1.16x fits the set of data, which indicates the relative increment relationship between Young’s modulus and shore hardness together. Ecoflex mixture ratio 4:0:0:1 (0010:0020:0030:Gel2) shows the highest shore hardness ($\bar{SH}$ = 26) and stiffness (30 kPa) compared to all data sets, which emphasize the effect of polymer chain structure and crosslinking agent on the material properties, while individual Ecoflex 0010 and Gel2 had lesser shore hardnesses (Table 2).

**Figure 17:**
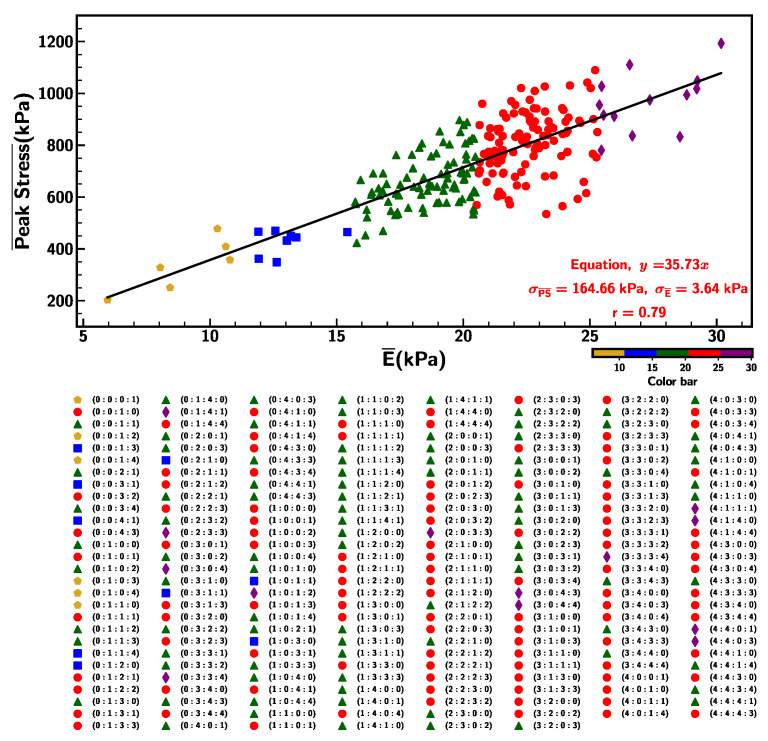
Correlation between Young’s modulus and peak stress at strain rate of 0.1/s.

On the contrary, the relationship between peak stress and shore hardness demonstrates a relatively weaker correlation (r = 0.52), as represented by Figure 16b. Each range of shore hardness in the data set represents the unique cluster of the Ecoflex mixture, highlighting the categorical behavior of data points (shown in color labels). Additionally, the proximity of Young’s modulus and maximum stress at strain 1200% was evaluated and is depicted in Figure 17. The correlation was found to be 0.79 and the equation y=35.73x signifies the stronger positive relationship among the two parameters.

### 3.8. Aging

**Figure 18:**
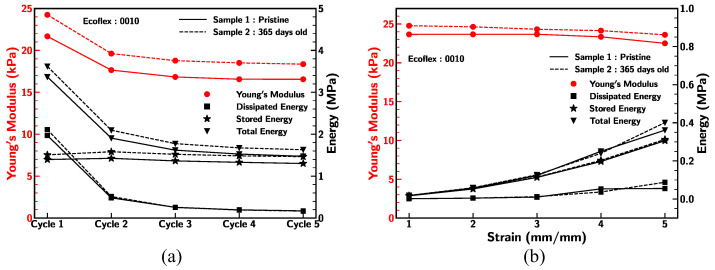
(**a**,**b**) Comparison of Young’s modulus, dissipated energy density, stored energy density, andtotal energy density for Ecoflex 0010 sample 1 (pristine) and sample 2 (365 days old) at strain rate of 0.1/s under different conditions.

The aging effect of Ecoflex 0010 was studied by subjecting the samples under five cyclic loading–unloading conditions and the Mullins effect as per above Section 3.4 and Section 3.5. The outcomes of sample 1 and sample 2 were depicted in Figure 18. Sample 1 (pristine) and sample 2 (365 days old sitting) were prepared from the same solution and sample 2 was stored in the laboratory at room temperature and normal condition. Both samples exhibited similar behavior of reduction in stiffness and energy density under identical cyclic loading–unloading conditions. Comparable characteristics were observed for sample 1 and sample 2 for Young’s modulus and energy density under the Mullins effect.

Both samples demonstrated comparable stiffness (13% error) and reduction in energy density (5% error) behavior when subjected to the identical cyclic loading–unloading condition. Also, samples 1 and 2 showed similar characteristics in terms of Young’s modulus (5% error) and energy density under the Mullins effect. This result suggests that the durability and performance of Ecoflex 0010 samples were not significantly impacted by aging and storage conditions.

**Figure 19:**
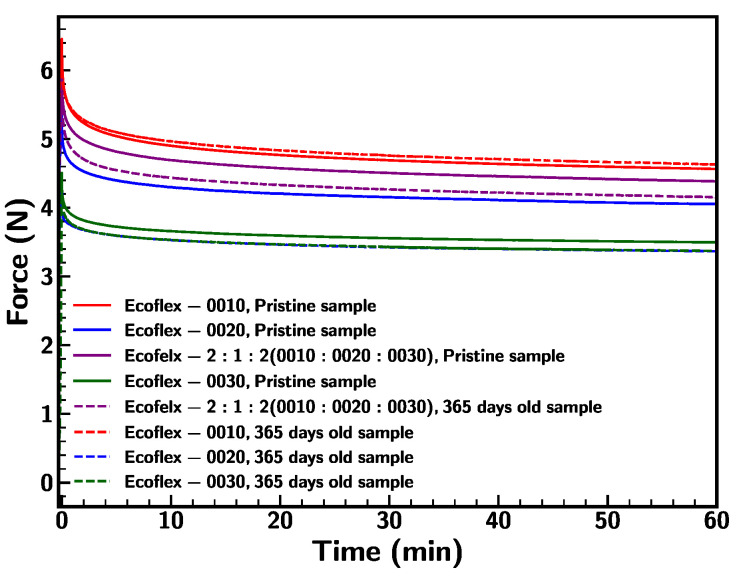
Comparison of stress–strain behavior of pristine and 365 old base samples at strain rate of 0.1/s.

Further, the durability of the base samples was investigated by subjecting the samples under stress relaxation at a strain of 1200% and the results are demonstrated in Figure 19. Both pristine and 365 days old samples exhibited relatively similar behavior with respect to achieving the steady state. Among all, the old Ecoflex 0020 sample responded to lower resistance (relaxation ratio = 24% and stress = 480 kPa) with respect to the stretch condition in comparison to the pristine sample (relaxation ratio = 22% and stress = 590 kPa). The rest of the base samples showed slight changes to the relaxation ratio and resistance with respect to the pristine sample. In summary, Ecoflex samples were less impacted by the environmental conditions.

### 3.9. Surface Morphology

**Figure 20:**
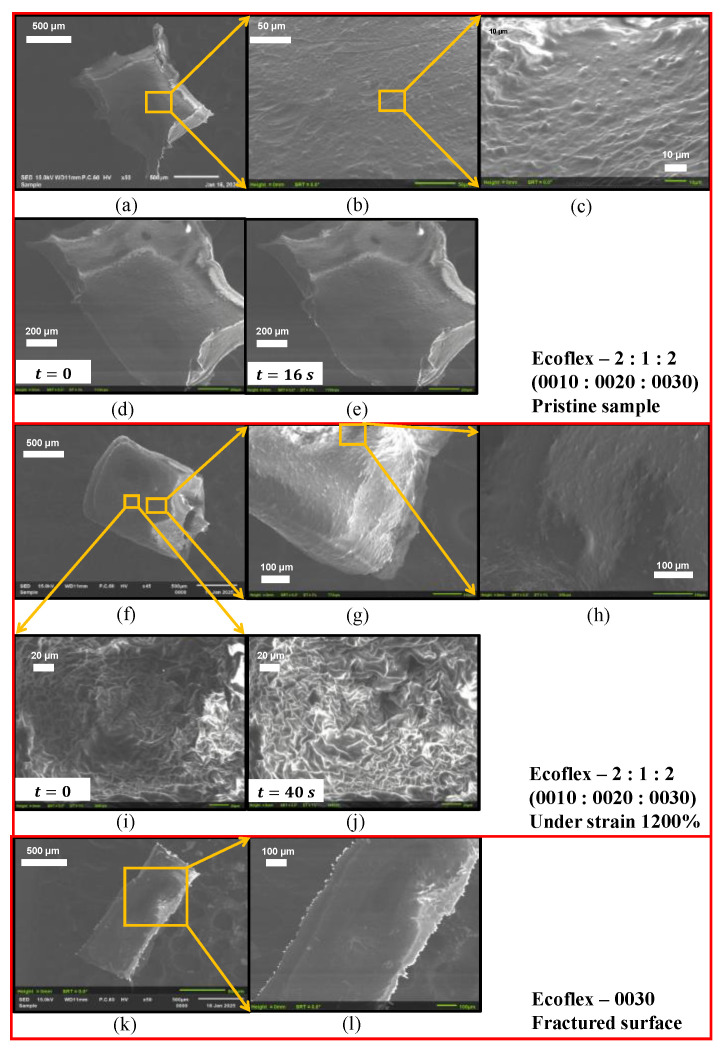
SEM images of Ecoflex samples (**a**–**e**) pristine 2:1:2 (0010:0020:0030) and (**f**–**j**) 2:1:2 (0010:0020:0030) under strain 1200% and (**k**,**l**) 0030 fractured.

The surface morphology of various Ecoflex samples under different conditions was investigated using SEM, as illustrated in Figure 20. The surface texture of the pristine and stretched Ecoflex sample 2:1:2 (0010:0020:0030) (Figure 20a–c,f–h) and 0030 (Figure 20k–l) reveals a smooth, intact surface with no visible differences or visible damage under SEM (resolution 10 µm). Figure 20d–e,i–j and Supplementary Videos S1 and S2 illustrate the conformational variations observed in the stretched Ecoflex sample under the influence of SEM analysis. Observation revealed that the stretched Ecoflex 2:1:2 sample exhibited a higher susceptibility to deformation compared to the pristine Ecoflex 2:1:2 sample (Figure 20d–e), which suggests that the softening of the sample was due to stretching (from earlier Section 3.4). The recovery of the surface texture of Ecoflex 0030 was observed upon removal of the focused beam of electrons, which signifies the adaptability of the samples to environmental changes, as demonstrated in Supplementary Video S3.

**Figure 21:**
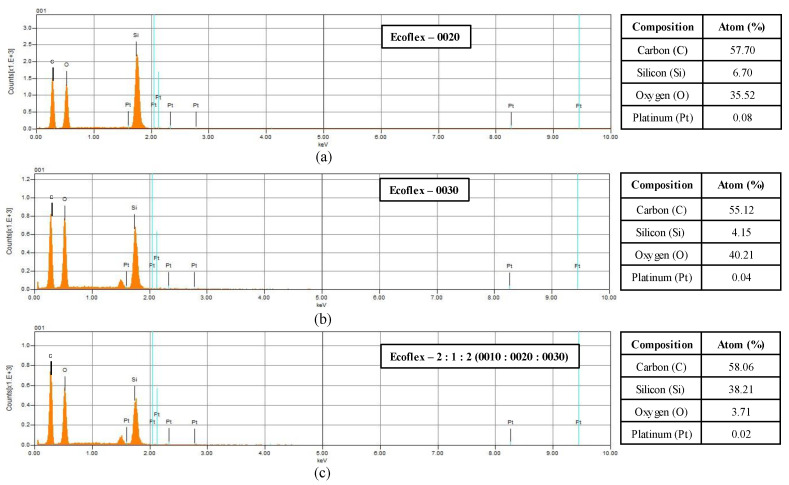
Energy Dispersive X-ray Spectroscopy (EDS) spectra and the atomic percentages of the main elements (C, Si, Pt, and O) summarized in tables for Ecoflex (**a**) 0020, (**b**) 0030, and (**c**) 2:1:2 (0010:0020:0030).

For the elemental atomic percent of elements in the Ecoflex samples, Energy Dispersive X-ray Spectroscopy (EDS) spectra analysis was evaluated, as presented in Figure 21 with summarized tables. EDS analysis confirmed the presence of elements silicon (Si) and oxygen (O), indicating the possible representation of siloxane (Si-O) compounds. Additionally, carbon (C), a component of methyl groups–(${CH}_{3}$), was also detected [49]. This resembles the results evaluated through FTIR from previous studies [61]. Platinum (Pt) element is likely to be anticipated from the Pt-based crosslinker. Variation in the mass composition of the sample (Figure 21) signifies the potential influence on the material properties such as flexibility, curing, and softness of the Ecoflex. However, results from Section 3.3 (strain rate) suggested that the final mechanical response of the Ecoflex samples mainly depends on the complex behavior of the polymer chain such as entanglements, chain slipping, dangling chains, and bond rupture [62,63].

### 3.10. Hydrophobicity

This section explores the method to improve the non-adhesiveness and smoothness of the Ecoflex surface. The sample was prepared by adding surface tension diffuser (ST-Diffuser) in varying weight percentages (x = 0 wt%, 0.15 wt%, 1.5 wt%, and 15 wt%) relative to the total amount of Ecoflex 0020. Probe TA–3 (diameter 25.4 mm) was utilized for measuring the stickiness of the sample with a load cell speed of 1 mm/s for a depth of 3.5 mm.

**Figure 22:**
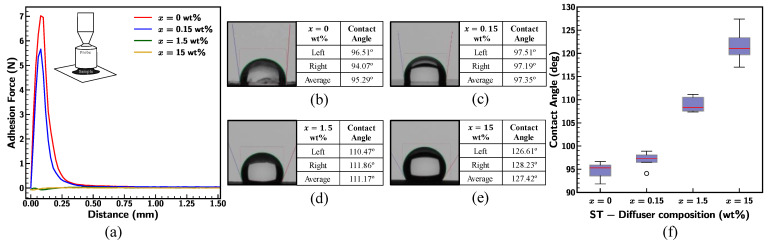
(**a**) Comparison of adhesion force; (**b**–**e**) contact angle of individual droplet summarized in tables; (**f**) variation in average contact angle of Ecoflex 0020 for varied ST-Diffuser composition.

The experimental data showed that the addition of ST-Diffuser concentration improves the non-adhesiveness of the Ecoflex sample, as presented in Figure 22a. Stickiness was found highest (7 N) for the pristine 0020 sample and the surface turned into a non-adhesive for x = 1.5 wt% and 15 wt%. In addition, hydrophobicity of the surface was measured using deionized (DI) water and the results are illustrated in Figure 22b–f. The mean contact angle was measured by averaging the value of both the left and right edges of the individual droplet, as shown in Figure 22b–e. Although previous adhesion test data indicated that the pristine Ecoflex 0020 (x = 0 wt%) was sticky, still it repels the water, showing the behavior of the hydrophobic surface. Blending the ST-Diffuser composition to the Ecoflex enhances the surface hydrophobicity with an increase in concentration. Average measured values were determined to be 122° for x = 15 wt% (Figure 22f), implying the ability to use as self-cleaning surfaces.

**Figure 23:**
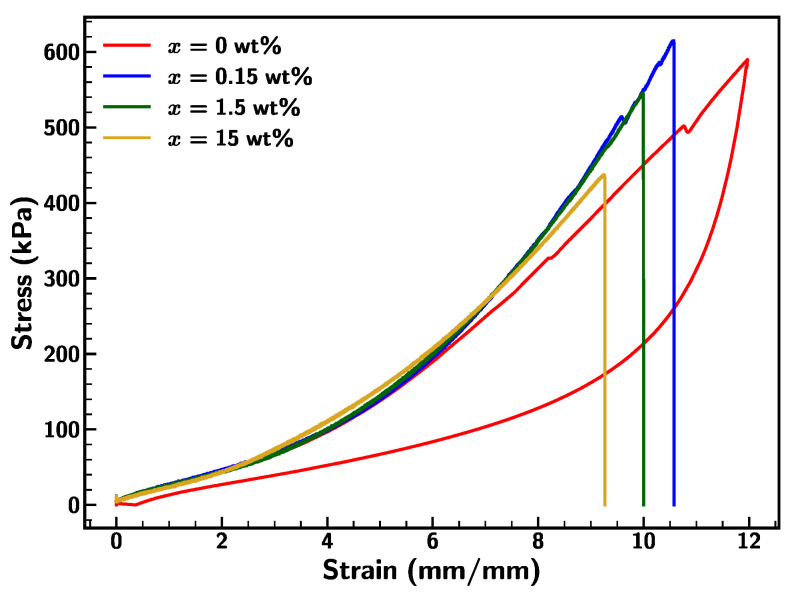
Stress–strain curve for Ecoflex 0020 with varying ST-Diffuser concentration at strain rate of 0.1/s.

Further, the impact of ST-Diffuser concentration on the mechanical behavior of Ecoflex 0020 was studied. The outcomes reflect the decrement in the stretchability of 0020 with the ST-Diffuser additive composite, as shown in Figure 23. The mechanical failure was observed for x = 15 wt% at 920% strain with least stretchability in comparison to other concentrations. Additionally, resistance from the samples was increased for larger deformation (>500%), indicating the stiffening of the material before fracture. Overall, ST-Diffuser additives improve the non-adhesive, hydrophobicity, and smoothness of the Ecoflex surface, though there was a trade-off in the stretchability, material behavior under large strain, and changes in the specimen color.

### 3.11. Simulation

Uniaxial tensile test experiments described in the above section were simulated using COMSOL Multiphysics software (v. 6.1) [64,65] to understand the stress distribution in the dogbone specimen. The discretized FEM domain consists of 16971 tetrahedral elements. The left end of the domain was subjected to a fixed boundary condition, while a strain rate was applied to the right end by adhering to the experimental condition. The Yeoh material model was adopted to simulate the large strain (up to 1200% strain), and Table 3 shows the material parameters used for the simulation (Supplementary Tabel S1 provides a detailed description). Time-dependent simulations were performed by varying the stretching speed according to the experimental condition.

**Figure 24:**
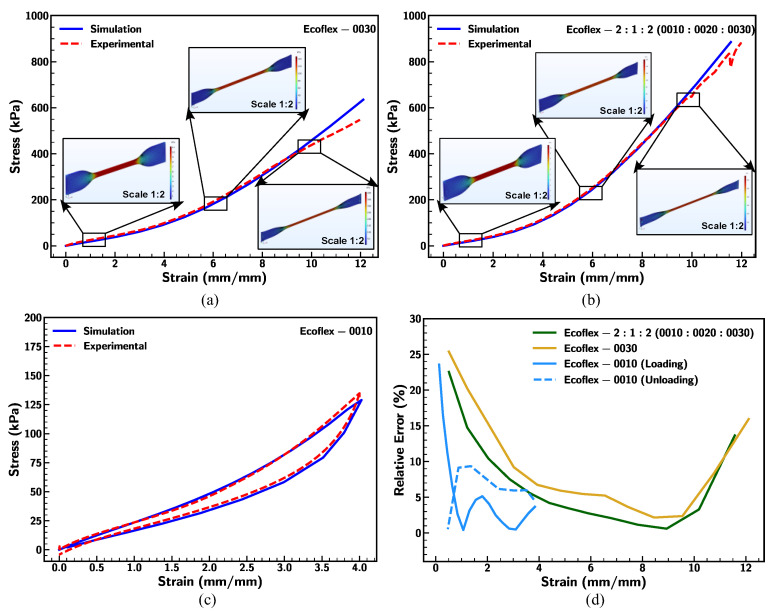
Comparison of simulation and experimental results of Ecoflex (**a**) 0030, (**b**) 2:1:2 (0010:0020:0030), and (**c**) 0010 and (**d**) relative error for strain rate of 0.1/s.

The simulation results of Ecoflex 0030 were in good agreement with the experimental data under larger strain, as demonstrated in Figure 24a. The respective stress distribution at 100% strain shows that the uniform stress was distributed across the gauge length of the dogbone specimen. Furthermore, stretching (at 600% strain) of the specimen induced deformation of the specimen with higher stress concentration along the gauge length. At large strain (1000% strain), the shape of the grip section changes, showing elongation of the material, with the risk of failure developing at the narrower section of the gauge length, which corresponds to the experimental failure observed in the specimen (shown in Figure 5e). Additionally, the Yeoh model was implemented to investigate the stress–strain behavior of the blending solution of Ecoflex 2:1:2 (0010:0020:0030). The simulation results showed that the material response of the blended solution agreed with the experimental results (shown in Figure 24b). In summary, Ecoflex and its mixing solution closely resemble the behavior of rubber-like materials along with softness, high flexibility, and stretchability.

In addition, the Yeoh model was adopted along with five terms of the Prony series to replicate the loading–unloading cycle under smaller strain to replicate the hysteretic behavior. The outcome of the simulation analysis for Ecoflex 0010 under strain = 400% is presented in Figure 24c. The simulation results follow closely with experimental results along with a reasonable representation of the energy dissipated in Ecoflex under cyclic loading–unloading conditions. The relative error was observed to be within 25% between simulation and experimental results, which decreases along with the stretching of the sample (up to 900% strain), as shown in Figure 24d. Overall, the applied Yeoh material model effectively predicts the mechanical behavior of the Ecoflex and its formulation, which can be further implemented for the characterization of the Ecoflex or silicone elastomer-like material.

## 4. Conclusions

This work presents a comprehensive experiment, simulation, and characterization of Ecoflex and its formulation based on weight concentrations subjected to monotonic uniaxial testing. The study showed that Ecoflex 0030 (*SH* = 30) transformed into a softer material under large deformation (>400%) compared to Ecoflex 0010 (*SH* = 11.5). The results from different loading conditions displayed the tunability nature of the Ecoflex (4:1 (0010:Gel2)), with improved stiffness and ultimate strength of the material. This signifies that mixing multiple formulations of Ecoflex–Ecoflex composite can be used to tune the mechanical properties for specific applications such as artificial skin/tissue. The durability test between the pristine and 365 days old samples indicated a minor effect on the material response to the mechanical deformation, which emphasizes the insignificant influences on the material in ambient conditions. The addition of ST-Diffuser improved the non-adhesive and smooth texture of the surface, yielding an average contact angle of 122° for Ecoflex 0020 at x= 15 wt% of additives. Furthermore, enhancing the hydrophobicity of the surface can be applied to create a soft touch, moisture-resistant, self-cleaning wearable devices. The adopted Yeoh model predicts the viscoelastic behavior and hysteresis of the Ecoflex and its blended composite, with a 25% relative error for large deformation (1200% strain) under loading and unloading cycles. The detailed analyses and databases allow researchers to select multiple formulations of Ecoflex–Ecoflex composite for their desired applications. Future work will focus on the integration of machine learning for the optimization of mixing formulation and prediction of mechanical properties.

## Figures and Tables

**Table 1:** Stress relaxation for base Ecoflex samples at strain rate of 0.1/s.

Ecoflex	Relaxation Ratio (%)			
Strain	200%	600%	1000%	1200%
0010	3.8	12.7		29.1
0020	3.7	13.7		24.1
0030	2.8	11.7	20.1	
Gel2	407	7.4		24.7
2:1 (0010:0020)				28.9
2:1:2 (0010:0020:0030)				26
2:1:2:2 (0010:0020:0030:Gel2)				24.4

**Table 2:** Measurement of shore hardness for base samples.

Ecoflex	$\bar{SH}$
0010	11.5
0020	19.85
0030	25
Gel2	5.05
2:1 (0010:0020)	18.4
2:1:2 (0010:0020:0030)	16.2
2:1:2:2 (0010:0020:0030:Gel2)	17.55

**Table 3:** Material properties.

**Yeoh Material Model**	**Ecoflex 0030**	**Ecoflex 2:1:2 (0010:0020:0030)**
$C_{1}$ (MPa)	0.0033	0.0031
$C_{2}$ (MPa)	0	0
$C_{3}$ (MPa)	$1.17\times 10^{-5}$	$2\times 10^{-5}$
κ (MPa)	0.78	1.13
υ	0.4	0.4

## Data Availability

The original contributions presented in this study are included in the article/Supplementary Material. Further inquiries can be directed to the corresponding author.

## Supplementary Materials

The following supporting information can be downloaded at: https://www.mdpi.com/article/10.3390/ma18133037/s1. Table S1. Detailed simulation setup and material model. Table S2. Detailed weight concentration of Ecoflex and its formulation solution. Figure S1. (a–f): Comparison of Young’s modulus and peak stress of Ecoflex and its formulation. Figure S2. (a–d): Comparison of stretchability of Ecoflex and its formulation. Video S1. Pristine_Ecoflex_2_1_2_under_SEM. Video S2. Streched_Ecoflex_2_1_2_under_SEM. Video S3. Recovery_of_Ecoflex_0030_under_SEM. Video S4. Vibratiion_of_Ecoflex_0020_under_SEM. Video S5. Simulaton_loading_unloading_strain_400.gif.
