# Supplementary material for: A Comprehensive Experimental, Simulation, and Characterization Mechanical Analysis of Ecoflex and Its Formulation Under Uniaxial Testing
Source: Materials (Basel). 2025 Jun 26;18(13):3037. doi: 10.3390/ma18133037 (PMC12251012; doi:10.3390/ma18133037)
Supplement: Supplementary file 1 [file materials-18-03037-s001.zip › Supplementary Materials.pdf]

Supporting Information

A Comprehensive Experimental, Simulation, and Characterization Analysis of Ecoflex and its formulation under Uniaxial Testing

Ranjith Janardhana[a], Fazli Akram[a], Zeynel Guler[a], Akanksha Adaval[a] and Nathan Jackson[a,b,*]

[a]*The Department of Mechanical Engineering and Center for High Technology Materials, The University of New Mexico, Albuquerque, NM 87131, USA*

[b]*The Nanoscience and Microsystems Engineering, The University of New Mexico, Albuquerque, NM 87131, USA*

[*]Corresponding *E-mail*: njack@unm.edu (Nathan Jackson)

SI Simulation Setup and Material Model

Table S1. Detailed Simulation Setup and Material Model.

<table>
<tr><th></th><th>Ecoflex – 0030</th><th>Ecoflex – 2 : 1 : 2
(0010 : 0020 : 0030)</th><th colspan="2">Ecoflex – 0010</th></tr>
<tr><td colspan="5">Yeoh model</td></tr>
<tr><td>C_1 (MPa)</td><td>0.0033</td><td>0.0031</td><td colspan="2">0.0048</td></tr>
<tr><td>C_2 (MPa)</td><td>0</td><td>0</td><td colspan="2">0</td></tr>
<tr><td>C_3 (MPa)</td><td>1.17×10^{-5}</td><td>2×10^{-5}</td><td colspan="2">2.4×10^{-5}</td></tr>
<tr><td>κ (MPa)</td><td>0.78</td><td>1.13</td><td colspan="2">1.5</td></tr>
<tr><td>υ</td><td>0.4</td><td>0.4</td><td colspan="2">0.4</td></tr>
<tr><td>ρ (kg/ m^3)</td><td>970</td><td>970</td><td colspan="2">970</td></tr>
<tr><td colspan="5">Plasticity: Johnson-Cook model</td></tr>
<tr><td colspan="5">Default value</td></tr>
<tr><td colspan="5">Mullins Effect: Ogden-Roxburgh model</td></tr>
<tr><td>d_∞</td><td>0.9</td><td>0.9</td><td colspan="2">0.3</td></tr>
<tr><td>$W_{m,sat}$ (J/m^3)</td><td>1×10^6</td><td>1×10^6</td><td colspan="2">6×10^3</td></tr>
<tr><td>$\propto$</td><td>0</td><td>0</td><td colspan="2">8×10^{-6}</td></tr>
<tr><td colspan="3">Shift Function: Williams-Landel-Ferry</td><td colspan="2">None</td></tr>
<tr><td colspan="5">Viscoelasticity: Generalized Maxwell</td></tr>
<tr><td colspan="3" rowspan="5">$E_{f1} = 0.2$
$t_1 = 3000$</td><td>$E_{f1} = 0.27$</td><td>$t_1 = 0.01$</td></tr>
<tr><td>$E_{f2} = 0.033$</td><td>$t_2 = 0.1$</td></tr>
<tr><td>$E_{f3} = 0.003$</td><td>$t_3 = 1$</td></tr>
<tr><td>$E_{f4} = 9 \times 10^{-5}$</td><td>$t_4 = 10$</td></tr>
<tr><td>$E_{f5} = 0.0006$</td><td>$t_5 = 100$</td></tr>
</table>

SI Weight Concentration of Ecoflex and its formulation

Table S2. Detailed weight concentration of Ecoflex and its formulation solution.

Total weight of solution in a mixing container: 22g									
Base liquid		Two type mixing		Three type mixing				Four type mixing	
Ecoflex shore hardness	Weight ratio	Ecoflex shore hardness	Weight ratio	Ecoflex shore hardness	Weight ratio	Ecoflex shore hardness	Weight ratio	Ecoflex shore hardness	Weight ratio
0010	1	0010:0020	1:1	0010:0020:0030	1:1:1	0010:0030:Gel2	1:1:1	0010:0020:0030:Gel2	1:1:1:1
0020	1		4:3		3:4:4		3:4:4		3:4:4:4
0030	1		2:1		1:2:2		1:2:2		1:2:2:2
Gel2	1		4:1		1:4:4		1:4:4		1:4:4:4
		0010:0030	1:1		4:3:4		4:3:4		4:3:4:4
			4:3		2:1:2		2:1:2		2:1:2:2
			2:1		4:1:4		4:1:4		4:1:4:4
			4:1		4:4:3		4:4:3		4:4:3:4
		0010:Gel2	1:1		2:2:1		2:2:1		2:2:1:2
			4:3		4:4:1		4:4:1		4:4:1:4
			2:1		4:3:3		4:3:3		4:4:4:3
			4:1		2:3:3		2:3:3		2:2:2:1
		0020:0010	1:1		1:3:3		1:3:3		4:4:4:1
			4:3		3:4:3		3:4:3		4:3:3:3
			2:1		3:2:3		3:2:3		2:3:3:3
			4:1		3:1:3		3:1:3		1:3:3:3
		0020:0030	1:1		3:3:4		3:3:4		3:4:3:3
			4:3		3:3:2		3:3:2		3:2:3:3
			2:1		3:3:1		3:3:1		3:1:3:3
			4:1		2:1:1		2:1:1		3:3:4:3
		0020:Gel2	1:1		3:2:2		3:2:2		3:3:2:3
			4:3		1:2:1		1:2:1		3:3:1:3
			2:1		2:3:2		2:3:2		3:3:3:4
			4:1		1:1:2		1:1:2		3:3:3:2
		0030:0010	1:1		2:2:3		2:2:3		3:3:3:1
			4:3		4:1:1		4:1:1		2:1:1:1
			2:1		3:1:1		3:1:1		3:2:2:2
			4:1		1:4:1		1:4:1		1:2:1:1
		0030:0020	1:1		1:3:1		1:3:1		2:3:2:2
			4:3		1:1:4		1:1:4		1:1:2:1
			2:1		1:1:3		1:1:3		2:2:3:2
			4:1	0010:0020:Gel2	1:1:1	0020:0030:Gel2	1:1:1		1:1:1:2
		0030:Gel2	1:1		3:4:4		3:4:4		2:2:2:3
			4:3		1:2:2		1:2:2		4:1:1:1
			2:1		1:4:4		1:4:4		3:1:1:1
			4:1		4:3:4		4:3:4		1:4:1:1
		Gel2:0010	1:1		2:1:2		2:1:2		1:3:1:1
			4:3		4:1:4		4:1:4		1:1:4:1
			2:1		4:4:3		4:4:3		1:1:3:1
			4:1		2:2:1		2:2:1		1:1:1:4
		Gel2:0020	1:1		4:4:1		4:4:1		1:1:1:3
			4:3		4:3:3		4:3:3		
			2:1		2:3:3		2:3:3		
			4:1		1:3:3		1:3:3		
		Gel2:0030	1:1		3:4:3		3:4:3		
			4:3		3:2:3		3:2:3		
			2:1		3:1:3		3:1:3		
			4:1		3:3:4		3:3:4		
					3:3:2		3:3:2		
					3:3:1		3:3:1		
					2:1:1		2:1:1		
					3:2:2		3:2:2		
					1:2:1		1:2:1		
					2:3:2		2:3:2		
					1:1:2		1:1:2		
					2:2:3		2:2:3		
					4:1:1		4:1:1		
					3:1:1		3:1:1		
					1:4:1		1:4:1		
					1:3:1		1:3:1		
					1:1:4		1:1:4		
					1:1:3		1:1:3		

SI Comparison of Young’s Modulus and Peak Stress

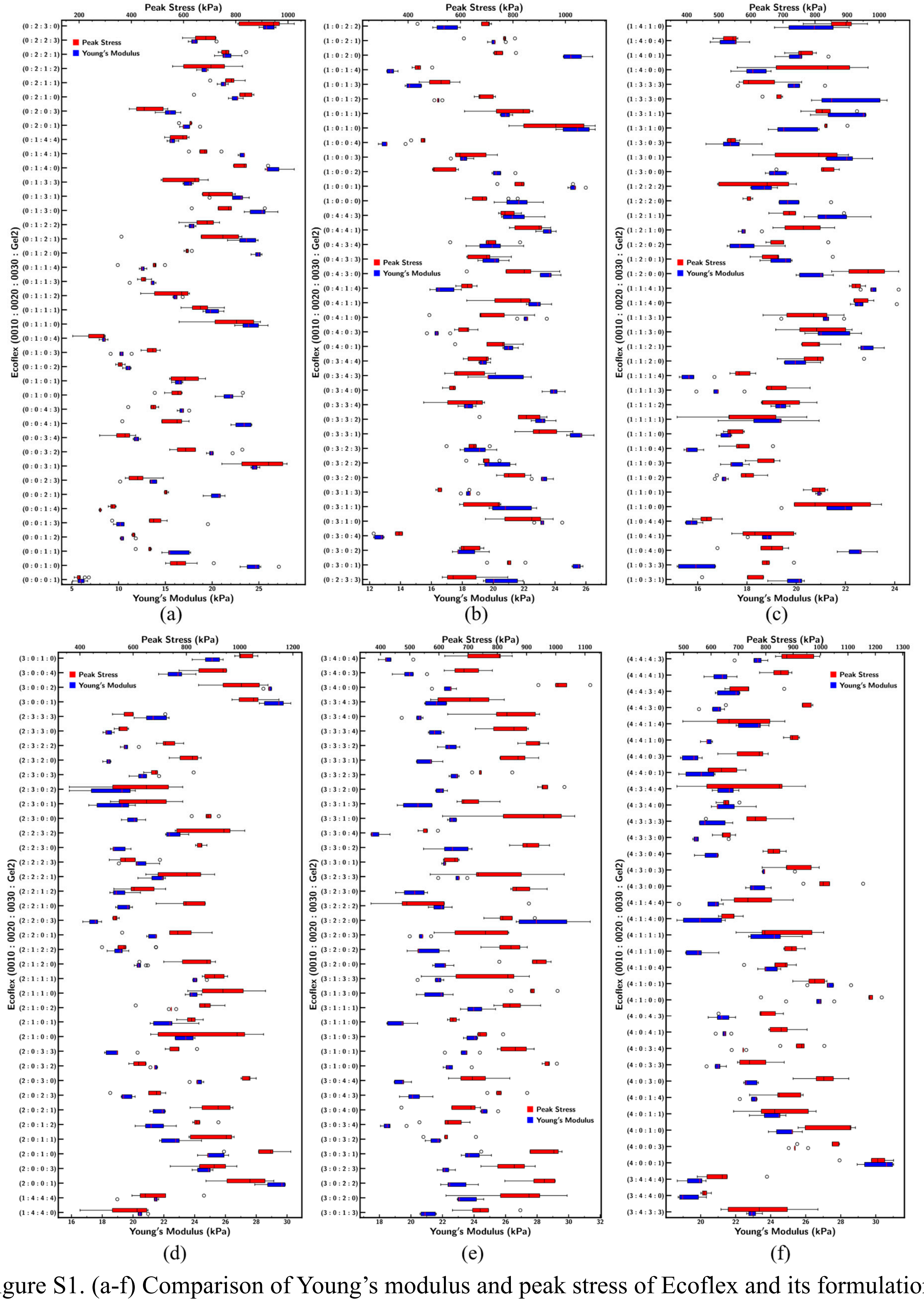

Figure S1. (a-f) Comparison of Young’s modulus and peak stress of Ecoflex and its formulation.

SI Stretchability

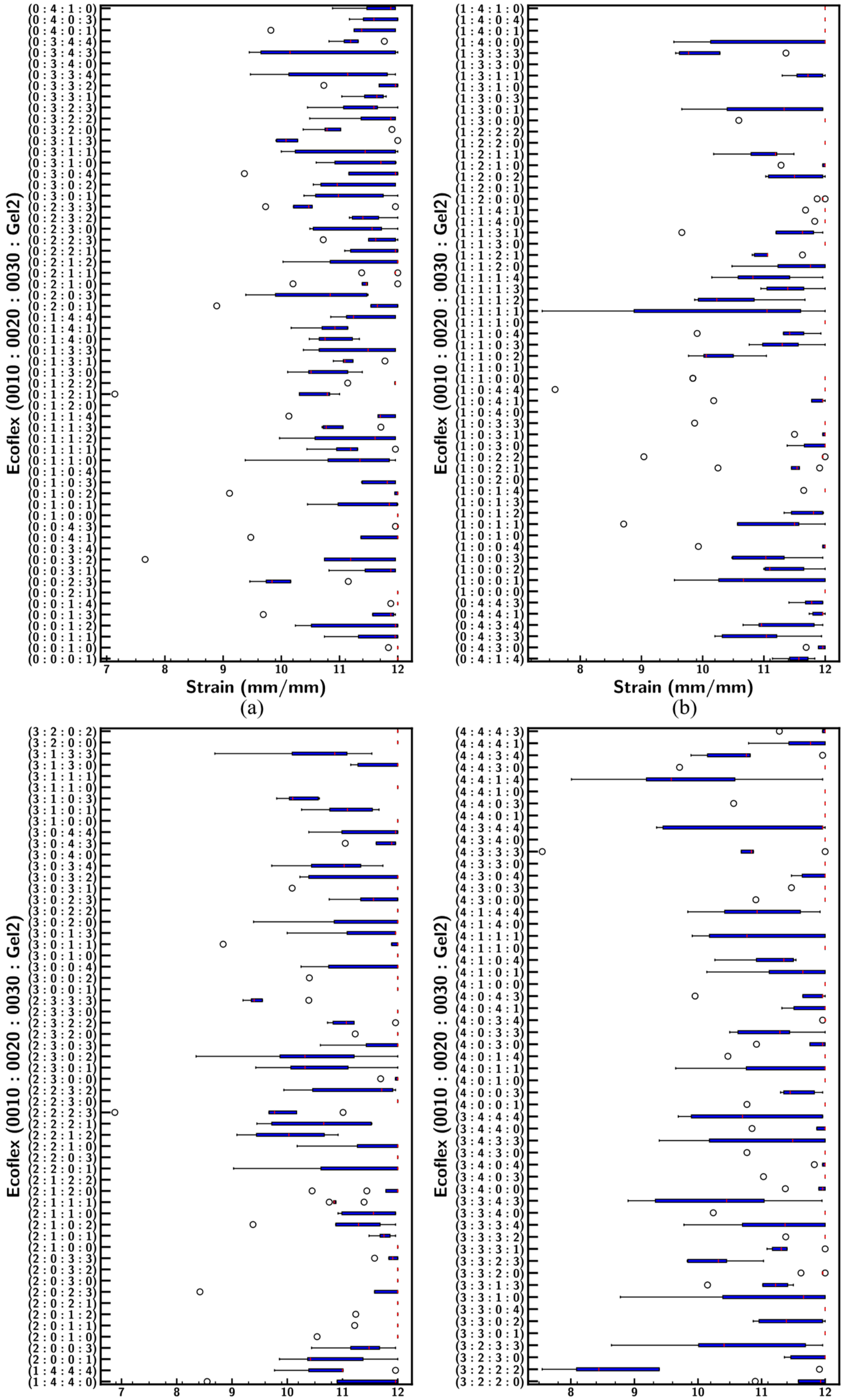

Figure S2. (a-d) Comparison of stretchability of Ecoflex and its formulation.
