# Supplementary figures and images for: A Comprehensive Experimental, Simulation, and Characterization Mechanical Analysis of Ecoflex and Its Formulation Under Uniaxial Testing
Source: Materials (Basel). 2025 Jun 26;18(13):3037. doi: 10.3390/ma18133037 (PMC12251012; doi:10.3390/ma18133037)

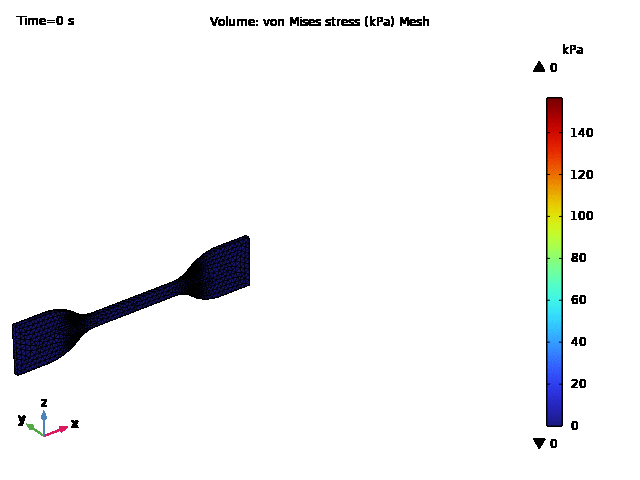

Supplement: Supplementary file 1 [file materials-18-03037-s001.zip › Video_S5_Simulaton_loading_unloading_strain_400.gif]
